# Epigenetic clock and methylation studies in the rhesus macaque

**DOI:** 10.1007/s11357-021-00429-8

**Published:** 2021-09-06

**Authors:** Steve Horvath, Joseph A. Zoller, Amin Haghani, Anna J. Jasinska, Ken Raj, Charles E. Breeze, Jason Ernst, Kelli L. Vaughan, Julie A. Mattison

**Affiliations:** 1 Department of Human Genetics, David Geffen School of Medicine, University of California, Los Angeles, Los Angeles, CA USA; 2 Department of Biostatistics, Fielding School of Public Health, University of California, Los Angeles, Los Angeles, CA USA; 3 Center for Neurobehavioral Genetics, Semel Institute for Neuroscience and Human Behavior, Department of Psychiatry and Biobehavioral Sciences, David Geffen School of Medicine, University of California, Los Angeles, CA Los Angeles, USA; 4 Radiation Effects Department, Centre for Radiation, Chemical and Environmental Hazards, Public Health England, Chilton, , Didcot, UK; 5 Altius Institute for Biomedical Sciences, Seattle, WA USA; 6 Department of Biological Chemistry, University of California, Los Angeles, Los Angeles, CA 90095 USA; 7 Translational Gerontology Branch, National Institute on Aging Intramural Research Program, National Institutes of Health, 16701 Elmer School Rd., MD 20842 Dickerson, USA

**Keywords:** Epigenetic clock, DNA methylation, Rhesus monkey, Nonhuman primate

## Abstract

Methylation levels at specific CpG positions in the genome have been used to develop accurate estimators of chronological age in humans, mice, and other species. Although epigenetic clocks are generally species-specific, the principles underpinning them appear to be conserved at least across the mammalian class. This is exemplified by the successful development of epigenetic clocks for mice and several other mammalian species. Here, we describe epigenetic clocks for the rhesus macaque (*Macaca mulatta*), the most widely used nonhuman primate in biological research. Using a custom methylation array (HorvathMammalMethylChip40), we profiled *n* = 281 tissue samples (blood, skin, adipose, kidney, liver, lung, muscle, and cerebral cortex). From these data, we generated five epigenetic clocks for macaques. These clocks differ with regard to applicability to different tissue types (pan-tissue, blood, skin), species (macaque only or both humans and macaques), and measure of age (chronological age versus relative age). Additionally, the age-based human-macaque clock exhibits a high age correlation (*R* = 0.89) with the vervet monkey (*Chlorocebus sabaeus*), another Old World species. Four CpGs within the *KLF14* promoter were consistently altered with age in four tissues (adipose, blood, cerebral cortex, skin). Future studies will be needed to evaluate whether these epigenetic clocks predict age-related conditions in the rhesus macaque.

**Supplementary Information:**

The online version contains supplementary material available at 10.1007/s11357-021-00429-8.

## Introduction

The rising costs of healthcare have fueled a growing need to address the leading risk factor of most diseases and health conditions—age. As such, investigations into the mechanisms and causes of aging, as well as interventions that might ameliorate its effects, hold great promise for improving health. To meet this end, animal models that closely recapitulate human aging are essential. Rhesus macaques (*Macaca mulatta*) are the most widely used nonhuman primate in biomedical research and share over 92% DNA sequence homology with humans [1]. They have an average lifespan in captivity of approximately 27 years, maximal lifespan of 42 years, and experience aging processes that are very similar to humans. With these features, the rhesus macaque presents as an excellent subject for the understanding of aging in humans and also other closely related primate species [2, 3]. Despite these attractive features, the employment of rhesus macaques in such research remains modest. This is due to both the prohibitive cost of maintaining a colony and the relatively long lifespan of these primates [4].

These challenges, however, can be effectively addressed if accurate and robust biomarkers of age can be established. Such biomarkers would change the experimental endpoint from longevity (measure of time from birth to death) to that of a surrogate endpoint (e.g., a molecular readout of health). The application of biomarkers will greatly reduce the duration and cost of primate studies, while generating a much more meaningful understanding of why we age and provide the means to evaluate anti-aging interventions.

Here, we report the development of DNA methylation–based biomarkers of age, known as epigenetic clocks for the rhesus macaque. Epigenetic clocks combine methylation levels of multiple CpGs to estimate chronological age or mortality risk (reviewed in [5–7]). As such, we interpret epigenetic age as one of several indicators of biological age, recognizing that it is not the same as biological age. The first human clocks leveraged methylation array platforms that provide accurate quantitative measurements of methylation for thousands of specific CpGs in the genome. Human and mouse pan-tissue DNA methylation (DNAm) age estimators exhibited important characteristics for aging studies, namely application to all sources of DNA (from sorted cells, tissues, and organs) and across the entire age spectrum (from prenatal tissue to centenarians) [5, 8–10]. The discrepancy between DNAm age and chronological age (termed as “epigenetic age acceleration”) is predictive of all-cause mortality in humans even after adjusting for a variety of known risk factors [11–13]. Several age-related conditions are also associated with epigenetic age acceleration, including, but not limited to, cognitive and physical functioning [14], centenarian status [13, 15], Down syndrome [16], HIV infection [17], and obesity [18].

Mouse epigenetic clocks accurately measure chronological age and have been successfully applied to confirm benchmark longevity interventions such as calorie restriction and ablation of growth hormone receptor [9, 10, 19–22]. While mouse clocks have not yet been correlated with their lifespan, it is expected that mortality risk prediction is not the preserve of human clocks but a feature that applies to several mammalian species. Although the human pan-tissue clock can be applied to chimpanzee DNA methylation profiles, its performance with profiles of other animals decline as a result of evolutionary genome sequence divergence [8]. Here, we describe the development and performance of several epigenetic clocks for rhesus macaques, two of which are dual-species clocks that apply both to humans and rhesus macaques.

## Brief methods and results

### DNA methylation data

All rhesus macaque DNA methylation profiles were generated on a custom methylation array (HorvathMammalMethylChip40) that measures the methylation level of 36,000 CpGs with flanking DNA sequences that are conserved across the mammalian class. We obtained 281 DNA methylation profiles from 8 different tissues of rhesus macaque (*Macaca mulatta*) with ages that ranged from 1.8 to 42 years (Table 1). An unsupervised hierarchical analysis clustered the methylation profiles by tissue type (Supplementary Fig. 1). DNA methylation–based age estimators (epigenetic clocks) were developed using data from *n* = 281 tissues, of which the most numerous were blood (*N* = 199) and skin (*N* = 51). Postmortem tissues (omental adipose, brain cortex, kidney, liver, lung, and skeletal muscle) were also available, but from fewer than 7 animals (Table 1). To generate dual-species epigenetic clocks that apply to both humans and rhesus macaques, *n* = 1207 human tissue samples were similarly profiled on the mammalian array platform (**Methods**).

**Table 1:** Description of rhesus tissues from which DNA methylation profiles were derived. *N*, total number of tissues. Number of females. Age: mean, minimum, and maximum in units of years

Tissue	*N*	No. of female	Mean.Age	Min.Age	Max.Age
Adipose	5	2	31.3	23.5	42
Blood	199	71	17.2	1.79	42
Cortex	6	3	29	17.2	42
Kidney	4	1	28.7	23.5	33.4
Liver	5	4	25.1	17.2	42
Lung	6	3	29	17.2	42
Muscle	5	2	30.1	17.2	42
Skin	51	13	18.8	7.61	42

### Epigenetic clocks

From these datasets, we generated five epigenetic clocks for macaques. These clocks differ with regard to applicability to different tissue types (pan-tissue, blood, skin), species (macaque only or both humans and macaques), and measure of age (chronological age versus relative age). As indicated by their names, pan-tissue clocks apply to all tissues, while the other clocks are developed for specific tissues/organs (blood, skin). The macaque pan-tissue clock was trained on all available tissues and applies only to rhesus macaques. The two human-macaque pan-tissue clocks, on the other hand, were derived from DNA methylation profiles from both species and are distinct from each other based on the unit of age that is employed. One estimates *chronological age* (in units of years), while the other estimates *relative* age, which is the ratio of chronological age to maximum lifespan, with values between 0 and 1. This ratio allows alignment and biologically meaningful comparison between species with very different lifespans (rhesus macaque and human), which is not afforded by mere measurement of chronological age. The maximum recorded lifespans for rhesus macaques and humans are 42 years and 122.5 years, respectively, according to the updated version of the *anAge* data base [23]; thus, there is an approximate 3:1 age ratio. By design, the human-rhesus clock for age does not account for differences in aging rates between species; i.e., an old macaque (~ 40 years old) is mathematically indistinguishable from a middle-aged human. By contrast, the human-rhesus clock for relative age implicitly accounts for differences in aging rates.

To arrive at unbiased estimates of the rhesus macaque pan-tissue clock, we carried out cross-validation analysis of the training data, followed by evaluation with an independent dataset from another nonhuman primate species (vervet monkey). The cross-validation study reports unbiased estimates of the age correlation *R* (defined as Pearson correlation between the DNAm age estimate and chronological age) as well as the median absolute error.

**Fig. 1:**
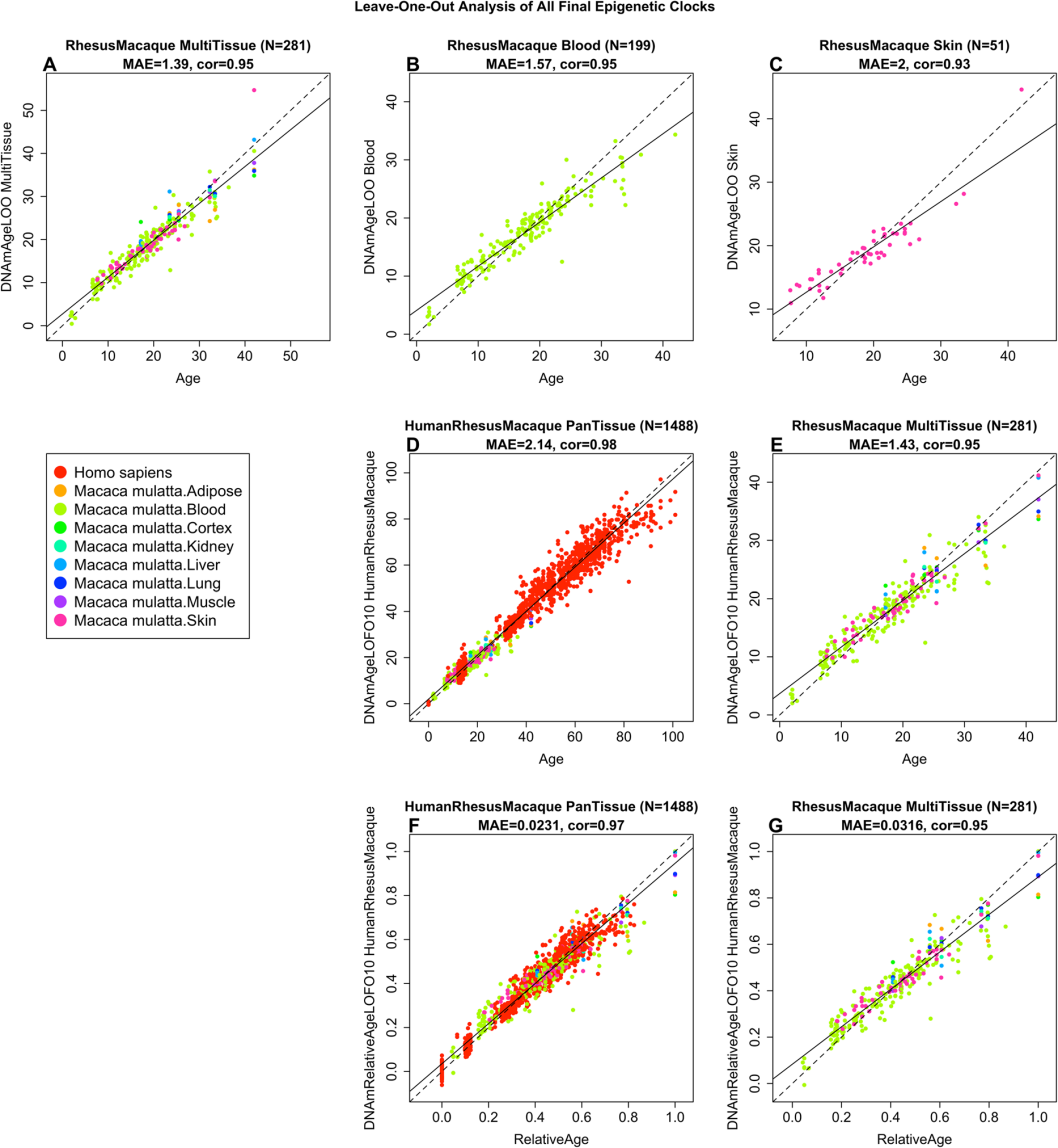
Cross-validation study of epigenetic clocks for rhesus macaques and humans. (**A**–**C**) Three epigenetic clocks that apply only to macaques. Leave-one-sample-out estimate of DNA methylation age (*y*-axis, in units of years) versus chronological age for (**A**) all available macaque tissues, (**B**) blood, and (**C**) skin. Ten-fold cross-validation analysis of the human-macaque monkey clocks for (**D**, **E**) chronological age and (**F**, **G**) relative age, respectively. (**D**, **F**) Human samples are colored in red and macaque samples are colored by macaque tissue type, and analogous in (**E**, **G**) but restricted to macaque samples (colored by macaque tissue type). Each panel reports the sample size (in parenthesis), correlation coefficient, and median absolute error (MAE)

**Fig. 2:**
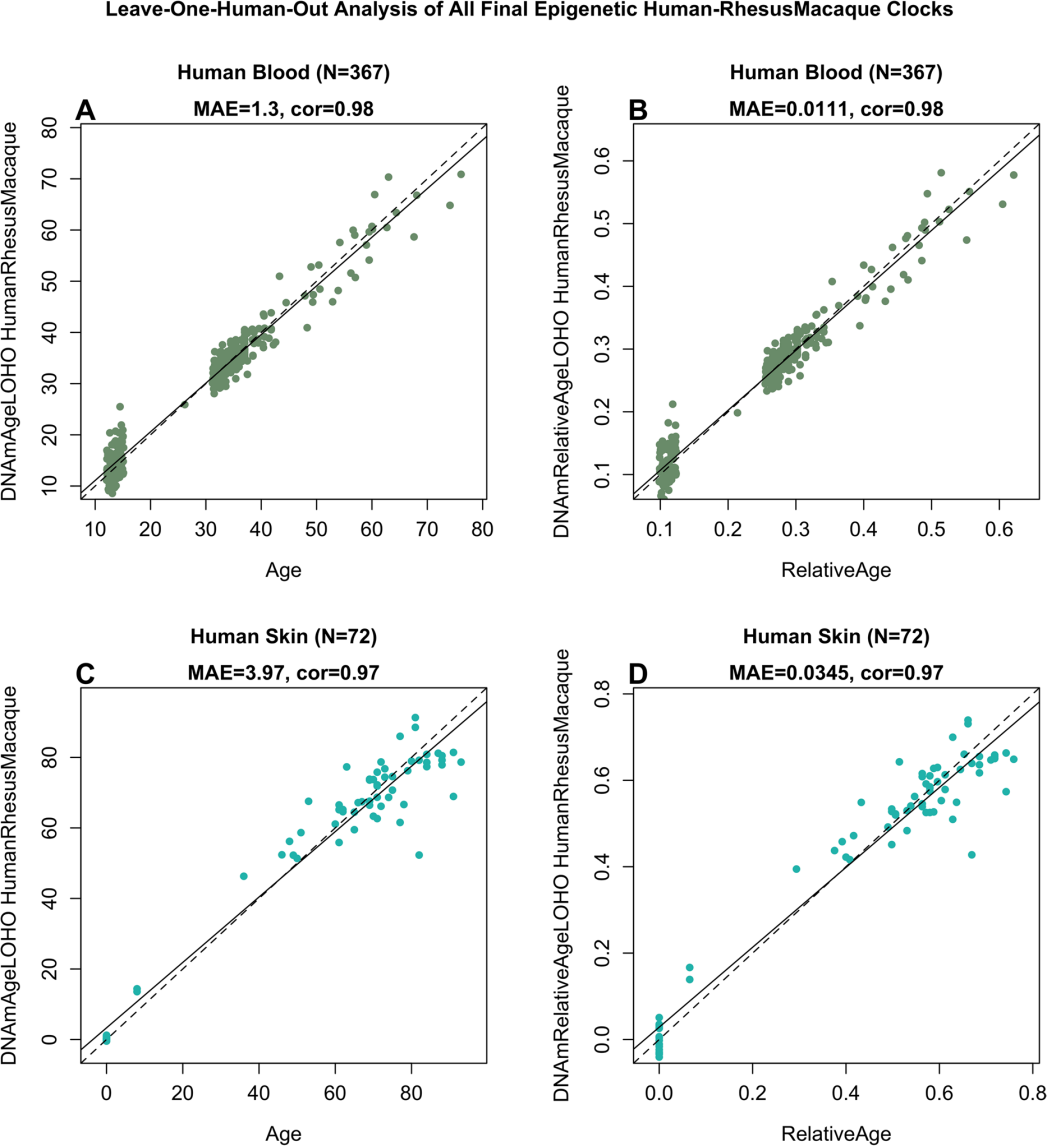
Human-macaque clocks applied to select human tissues. Leave-one-human sample-out (LOHO) cross fold cross-validation estimates of the human-macaque clock for (**A**, **C**) chronological age and (**C**, **D**) relative age, respectively. (**A**, **B**) Human blood samples. (**C**, **D**) Human skin samples. Each panel reports the sample size (in parenthesis), correlation coefficient, and median absolute error (MAE)

The resulting macaque pan-tissue clock is highly accurate in age estimation across tissues (*R* = 0.95, median absolute error (MAE) 1.4 years, Fig. 1A) and in individual types (*R* ≥ 0.93, Fig. 1B and C; Supplementary Fig. 2C–I), except for adipose tissue (*R* = 0.73, Supplementary Fig. 2B) for which only *n* = 5 samples were available. The human-rhesus macaque clock for age is highly accurate when both species are analyzed together (*R* = 0.98, Fig. 1D), with a slight reduction when the analysis is restricted to rhesus macaque tissues (*R* = 0.95, Fig. 1E). The human-rhesus macaque clock for *relative age* exhibits high correlation regardless of whether the analysis is done with samples from both species (*R* = 0.97, Fig. 1F) or with only rhesus macaque samples (*R* = 0.95, Fig. 1G). The employment of relative age circumvents the inevitable unequal distribution of data at the opposite ends of the age range when chronological age of species with very different lifespans is measured using a single formula. A cross-validation analysis reveals that both human-macaque clocks lead to high accuracy (*R* ≥ 0.97) in human blood and skin samples (Fig. 2).

### Cross-species performance of the rhesus macaque pan-tissue clock

**Fig. 3:**
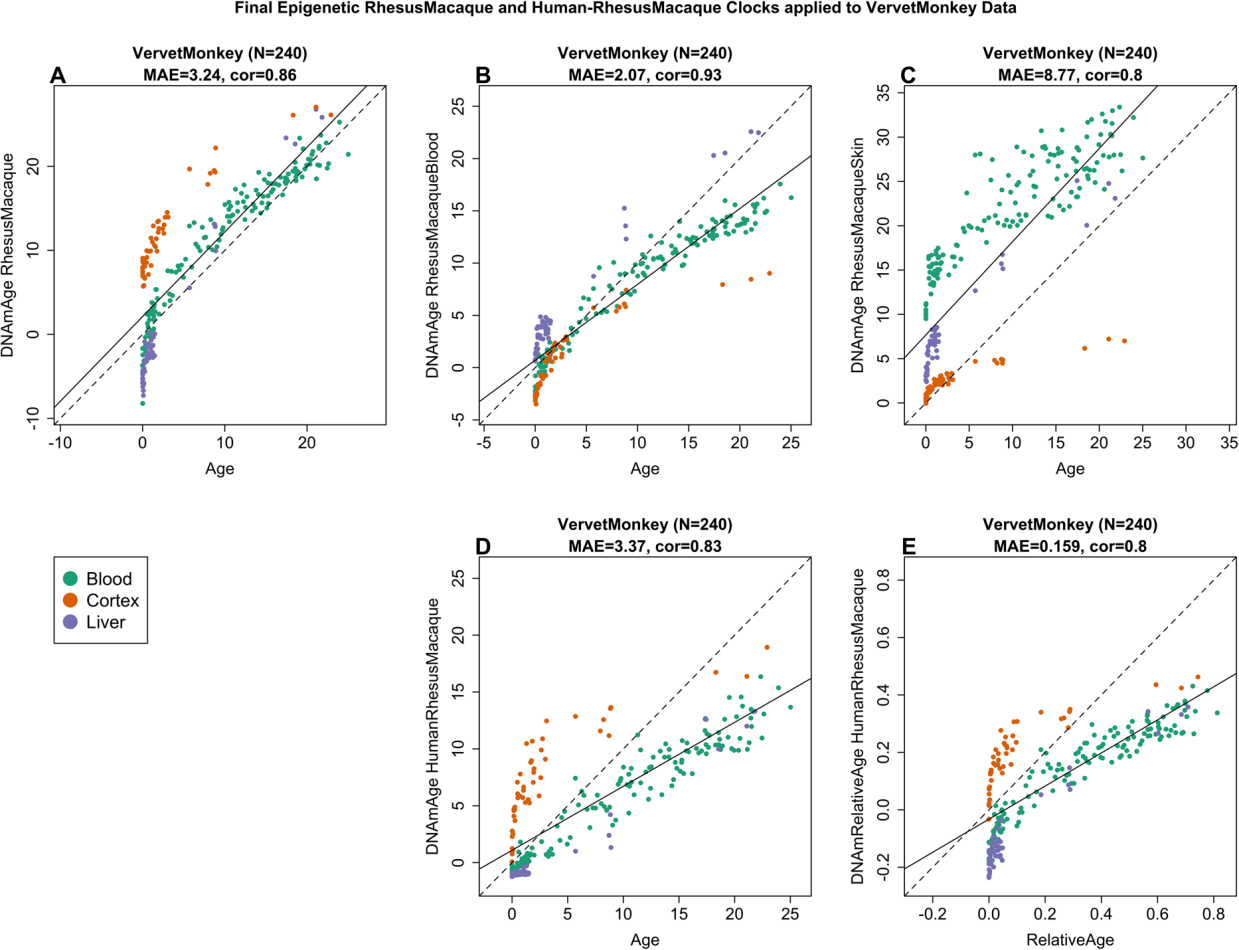
Macaque clocks applied to tissues from vervet monkey (*Chlorocebus sabaeus*). Each dot corresponds to a tissue sample from vervet monkeys. Each dot is colored by tissue type: blood (green), cerebral cortex (red), liver (purple). Chronological age of the vervet specimens (*x*-axis) versus the DNAm age estimate of the (**A**) pan-tissue macaque clock, (**B**) blood macaque clocks, (**C**) skin macaque clock, (**D**) human-macaque clock for chronological age, and (**E**) human-macaque clock for relative age. Each panel reports the sample size (in parenthesis), correlation coefficient, and median absolute error (MAE)

To determine the extent by which the rhesus macaque epigenetic clock can be applied to another primate, we used it to estimate the age of numerous tissues (blood, brain cortex, and liver) of the vervet monkey (*Chlorocebus sabaeus*), which is another Old World monkey separated 12.5 million years ago from the macaques. Despite this, we observed high correlations between the chronological age of vervets and their predicted age based on the macaque pan-tissue clock: *R* = 0.96 in vervet blood, *R* = 0.92 in vervet cortex, and *R* = 0.98 in vervet liver (Supplementary Fig. 3A-C, Fig. 3A–D). It is worth noting that the comparison of correlation coefficients between different tissues is not straightforward as these values are dependent on the age distribution of the samples that are evaluated (i.e.., minimum and maximum age). The rhesus pan-tissue and blood clocks work well in vervet blood but lead to substantial offset of 9 years in cerebral cortex (Supplementary Fig. 3B). Nevertheless, there is reasonably good concordance between chronological age of vervets and the estimated age of their blood (median error 1.9 years, Supplementary Fig. 3A) and liver (median error 3.7 years, Fig. 3C) by the macaque pan-tissue clock.

### Epigenome-wide association studies (EWAS) of chronological age in rhesus macaque

**Fig. 4:**
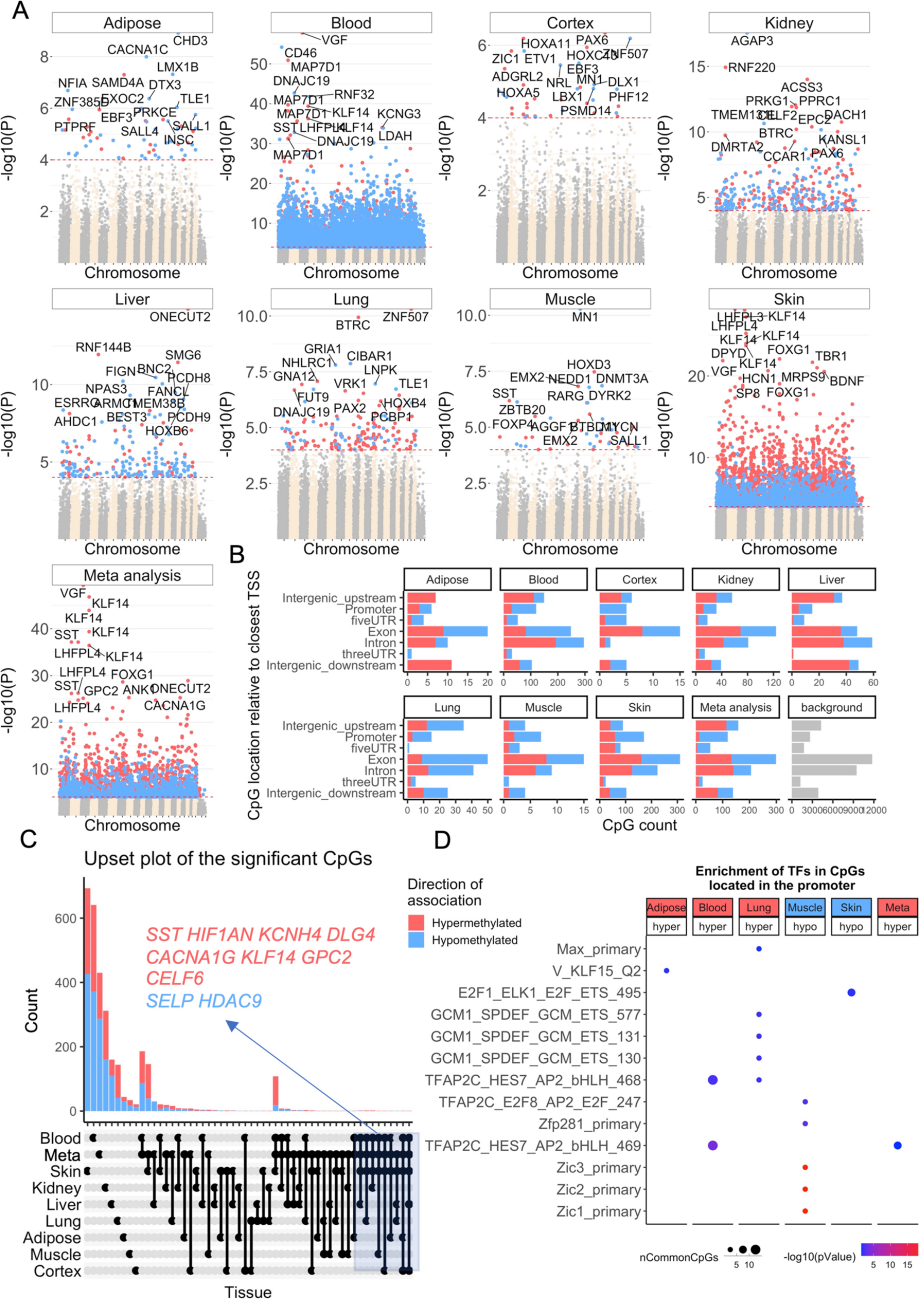
Epigenome-wide association studies (EWAS) of chronological age in adipose, blood, cerebral cortex, kidney, liver, lung, muscle, and skin of rhesus macaque. (**A**) Manhattan plots of the EWAS of chronological age. The coordinates are estimated based on the alignment of mammalian array probes to Mmul_10.100 genome assembly. The direction of associations with *p* < 10^−4^ (red dotted line) is highlighted by red (hypermethylated) and blue (hypomethylated) colors. Top 30 CpGs were labeled by the neighboring genes. (**B**) Location of top CpGs in each tissue relative to the closest transcriptional start site. Top CpGs were selected at *p* < 10^−4^ and further filtering based on *z* score of association with chronological age for up to 500 in a positive or negative direction. The number of selected CpGs: adipose, 62; blood, 1000; cerebral cortex, 40; kidney, 380; liver, 230; lung, 186; muscle, 47; skin, 1000; and meta-analysis, 1000. The gray color in the last panel represents the location of 36733 mammalian BeadChip array probes mapped to Mmul_10.100 genome. (**C**) Upset plot representing the overlap of age-associated CpGs based on meta-analysis or individual tissues. Neighboring genes of the overlapping CpGs were labeled in the figure. (**D**) Transcriptional motif enrichment for the top CpGs in the promoter and 5′UTR of the neighboring genes. The enrichment was tested using a hypergeometric test (**Methods**)

In total, 36,733 probes from HorvathMammalMethylChip40 could be mapped to specific loci in the rhesus macaque (*Macaca mulatta*.Mmul_10.100) genome. These loci are located proximal to 6154 genes. It is expected that output from the use of these clocks can be extrapolated to humans and other mammals since the mammalian array is designed to cover the most conserved regions across different mammalian genomes. To characterize the CpGs that change with macaque age (age-related CpGs) in different tissues, epigenome-wide association studies were carried out, which showed clear tissue specificity of age-related CpGs (and their proximal genes) (Fig. 4A). Hence, aging effects in one tissue do not appear to be reflected in another tissue (Supplementary Fig. 4). This, however, may be owed to the limited sample size in non-blood tissue (Table 1).

To identify CpGs whose methylation is most affected by age in all the tissues analyzed, DNAm changes were analyzed at a nominal *p* value < 10^−4^. The top DNAm changes and their proximal genes in each tissue are as follows: adipose, *CHD3* promoter (correlation test Z statistic *z* =  − 6); blood, *VGF* promoter (*z* = 16); cerebral cortex, *PAX6* 5′UTR (*z* = 5); kidney, *AGAP3* intron (*z* =  − 8.7); liver, *ONECUT2* exon (*z* = 7.9); lung, distal intergenic region upstream of *ZNF507* (*z* = 6.5), and *GRIA1* promoter (*z* =  − 5.7); muscle, *MN1* intron (*z* =  − 6.5); and skin, *LHFPL4* intron (*z* = 11). Meta-analysis of these eight tissues showed the top DNAm changes to include hypermethylation in *VGF* promoter (*z* = 14.8), four CpGs in *KLF14* promoter (*z* = 12.7 to 14.5), SST promoter (*z* = 12.9), and *LHFPL4* exon (*z* = 12.8) (Fig. 4A). CpGs that exhibited consistent age-associated methylation change across multiple (but not necessarily all) tissues were identified with an upset plot, which can be interpreted as a generalization of a Venn diagram. The upset plot analysis highlighted four CpGs in the *KLF14* promoter as being age-related in at least four tissues (adipose, blood, cortex, and skin, Fig. 4C). The *KLF14* promoter controls expression of the *KLF14* protein, which is itself a transcriptional factor that regulates the expression of TGFBII receptor.

Age-associated CpGs in different tissues were found to be distributed in genic and intergenic regions that can be defined relative to transcriptional start sites (Fig. 4B). However, in tissues with sufficient sample numbers (blood and skin), CpGs located in promoters and 5′UTRs had a higher percentage of DNAm change than the background. Moreover, the DNAm changes in promoter and 5′UTR were mainly hypermethylation in all tissues. This result paralleled prior observed patterns in DNAm aging in other species. We proceeded to identify putative transcriptional factors whose binding motifs were enriched for the top CpGs located in promoter or 5′UTR with DNAm changes, in either direction and in each tissue (Fig. 4D). The top TF motifs were Zic1 and Zic2, which had five CpGs that become less methylated with age in muscle. For blood and lung, the top enriched motif is the TFAP2C (AP-2 gamma transcriptional factor) binding site that becomes increasingly hypomethylated with age.

### Discussion

Human epigenetic clocks have many biomedical applications, including in human clinical trials [5, 24]. The utility of these human clocks prompted development of similar ones for other mammalian species. Clocks developed for mice are particularly important as they allow modelling of epigenetic age in a widely used model organism [9, 10, 19–22]. Despite the many advantages of mouse models, there is still a large gap in translating findings to primates. Hence, nonhuman primates play an indispensable role in preclinical investigations of potential interventions that might slow aging. As a case in point, both the National Institute on Aging and the University of Wisconsin have conducted longitudinal studies in rhesus macaques to determine if the promising anti-aging intervention, caloric restriction, would also apply to nonhuman primates and hence, more plausibly translate to human aging [25–27]. Indeed, these studies have yielded valuable information about the role of diet composition, fasting timing, and overall intake on healthspan and lifespan [26–28]. Despite their importance, such lifespan and healthspan studies in nonhuman primates are time consuming and costly. Therefore, the development of suitable biomarkers for aging promises to reduce the cost and time needed for carrying out such studies; thus, specific epigenetic clocks for nonhuman primate species are necessary. A critical step that obviates the species barrier was the development of a mammalian DNA methylation array that profiles up to 36,000 CpGs with flanking DNA sequences that are conserved across multiple mammalian species. This allows DNA methylation profiling of virtually all mammalian species. The rhesus macaque DNA methylation profiles detailed here were derived from eight tissue types and represent the largest dataset to date of single-base resolution methylomes in highly conserved regions across multiple tissues and ages.

This successful derivation of the multiple rhesus macaque epigenetic clocks attests to the conservation of epigenetic aging mechanisms across the mammalian class. The macaque clock exhibits impressive age correlation with the vervet monkey clock, a species which diverged 12.5 million years ago. Moreover, the evolutionary conservation of epigenetic aging is further exemplified by demonstrating the feasibility of combining methylation profiles of humans and rhesus macaque. These species diverged 29 million years ago, yet a single mathematical formula can be applied to generate human-rhesus macaque clocks. This single formula human-macaque clock is equally applicable to both species, and thereby demonstrates conservation of aging mechanisms, which alternatively could be deduced with the existence of multiple individual clocks for other mammals.

The significance of this unification under one formula has implications, which extend beyond its utility in directly translating age-related findings in rhesus macaques to humans. With this tool, one can consider the root contributions to aging as it affirms the increasing evidence that aging is a coordinated biological process, harmonized throughout the body. This ushers in the possibility that when a regulator or coordinator of aging rate is identified, there is potential to modulate it through interventions. As this mechanism is conserved across species, interventions that successfully alter the epigenetic aging rate of rhesus macaques, as measured using the human-rhesus macaque clock, will likely exert similar effects in humans.

Although genome- and epigenome-wide analyses often yield a large number of potential target genes and pathways related to aging, it is not immediately obvious which ones are actually relevant. Yet, with repeated analyses of age-related CpGs in different species within the mammalian class, the relevant candidates can be identified. By design, the mammalian array facilitates cross-species comparisons. As a case in point, analyses of datasets derived from this array revealed CpGs within the TFAP2 binding site were increasingly unmethylated with age across different mammalian species including the rhesus macaque. This motif is associated with genes that are involved in cell cycle arrest, germ cell development, and implicated in several types of cancers [29, 30]. Additionally, candidates such as Zic1 and Zic2, which did not feature in previously analyzed mammalian species, were uncovered and may indicate species-specific genes related to aging. These ZIC1 and ZIC2 transcription factors are particularly interesting because they regulate the expression of the *APOE* gene, which is associated with longevity and is the most commonly identified genetic risk factor of Alzheimer’s disease [31]. Thus, methylation change in this motif might underlie age-associated expression in this protein. Evolutionary selection and adaptation would predict a divergence in genes and pathways between species. This is akin to other biological processes, such as cell cycle regulation, where a basic mechanism is conserved across species, but special additions, deletions, and modifications are identified in only a select species or group.

Just as there are species differences, age-related DNA methylation changes are tissue specific. Sample size was a limitation of the current study, and thus we can draw only limited conclusions from our data presented here. This notwithstanding, it is interesting to note that CpGs within the KLF14 promoter were consistently altered with age in four tissues (adipose, blood, cerebral cortex, skin). KLF14 is a transcription factor that regulates the TGFBII receptor. This has potential physiological significance because the ligand of this receptor, TGFB, exerts diverse cellular effects including telomere regulation, unfolded protein response, autophagy, DNA repair, cellular senescence, and stem cell aging. As a consequence, TGFB signaling is frequently involved in age-related pathologies such as cardiovascular disease, Alzheimer’s disease, and osteoarthritis [32].

This is just one example from our extensive analysis of the rhesus epigenome that has broad tissue application and highlights the need for more in-depth empirical investigations to test and reveal the underlying mechanisms of epigenetic aging. Toward this end, the epigenetic clocks may play a pivotal role in uncovering potential candidates, monitoring aging rates, and testing putative aging interventions. The rhesus epigenetic clocks described here may be a key factor in translating such interventions to humans.

## Detailed methods

### Rhesus macaque

In total, we analyzed *N* = 281 rhesus macaque tissue samples from 8 different tissues (Table 1). The rhesus monkeys have been housed continuously at the NIH Animal Center, Poolesville, MD. The animal center is fully accredited by the American Association for Accreditation of Laboratory Animal Care, and all procedures were approved by the Animal Care and Use Committee of the NIA Intramural Program. Monkeys were of a heterogenous genetic background, both Chinese and Indian origin.

Monkeys were housed individually or paired in standard nonhuman primate caging on a 12-h light/12-h dark cycle, room temperature 78 ± 2° humidity at 60 ± 20%. Housing has been described previously [33]. All monkeys had extensive visual, auditory, and olfactory but limited tactile contact with monkeys housed in the same room. Monkeys received 2 meals per day at estimated ad libitum levels throughout the study. Water was always available ad libitum. Monkeys were monitored minimally 3 times daily by trained animal care staff.

### Sample collection

Monkeys were fasted overnight, approximately 16–18 h. Monkeys were anesthetized with either ketamine, 7–10 mg/kg, IM or Telazol, 3–5 mg/kg, IM. Blood samples were obtained by venipuncture of the femoral vein using a vacutainer and EDTA tubes. Samples were immediately placed on dry ice and stored at − 80°. Skin samples were collected at the same time from an alcohol-wiped area of the back between the shoulder blades.

Omental fat, kidney, liver, lung, skeletal muscle, and brain cortex were collected during necropsies scheduled for other study purposes or terminal clinical conditions. At that time, tissues were flash frozen in liquid nitrogen following collection and stored at − 80°. These tissues were selected for use based on having matching blood samples. None of the monkeys was sacrificed for this study.

### Vervet Monkeys

The vervet monkey data are described in a companion paper [34].

### Human tissue samples

To build the human-rhesus macaque clock, we analyzed previously generated methylation data from *n* = 1207 human tissue samples (adipose, blood, bone marrow, dermis, epidermis, heart, keratinocytes, fibroblasts, kidney, liver, lung, lymph node, muscle, pituitary, skin, spleen) from individuals whose ages ranged from 0 to 93 years. The tissue samples came from three sources: tissue and organ samples from the National NeuroAIDS Tissue Consortium [35], blood samples from the Cape Town Adolescent Antiretroviral Cohort study [36], skin and other primary cells provided by Kenneth Raj [37]. Ethics approval (IRB#15–001,454, IRB#16–000,471, IRB#18–000,315, IRB#16–002,028).

### DNA methylation profiling

All DNA methylation data were generated using the custom Infinium array “HorvathMammalMethylChip40” which uses both type I and type II infinium probes [38].

By design, the mammalian methylation array facilitates epigenetic studies across mammalian species (including rhesus macaques and humans) due to its very high coverage (over thousand-fold) of highly conserved CpGs in mammal [38]. Each probe is designed to cover a certain subset of species as detailed on our Github page. The chip manifest file can be found at Gene Expression Omnibus (GEO) at NCBI as platform GPL28271. Genome coordinates and other information can be downloaded from https://www.github.com/shorvath/MammalianMethylationConsortium/. The SeSaMe normalization method was used to define beta values for each probe [39].

### Penalized regression models

Details on the clocks (CpGs, genome coordinates) and R software code are provided in the Supplement. Our pan-tissue clock for rhesus macaque is based on 71 CpGs that are present on a custom chip (HorvathMammalMethylChip40). Our human-rhesus macaque epigenetic clock for chronological age is based on 508 CpGs. Another human-rhesus macaque epigenetic clock for relative age is based on 623 CpGs. We developed epigenetic clocks for rhesus macaques by regressing chronological age on the CpGs on the mammalian array. We used all tissues for the pan-tissue clock.

Penalized regression models were created with the R function “glmnet” [40]. We investigated models produced by both “elastic net” regression (alpha = 0.5). The optimal penalty parameters in all cases were determined automatically by using a tenfold internal cross-validation (cv.glmnet) on the training set. By definition, the alpha value for the elastic net regression was set to 0.5 (midpoint between Ridge and Lasso type regression) and was not optimized for model performance. We performed a cross-validation scheme for arriving at unbiased (or at least less biased) estimates of the accuracy of the different DNAm-based age estimators. One type consisted of leaving out a single sample (LOOCV) from the regression, predicting an age for that sample, and iterating over all samples.

For the cross-validation procedure, the penalized regression algorithm automatically selected a different set of CpGs from the array for each fold. In case of LOO cross-validation, the CpG selection was based on n-1 observations. A critical step is the transformation of chronological age (the dependent variable). While no transformation was used for the pan-tissue clock for rhesus macaque, we did use a log linear transformation for the dual species clock of chronological age (Supplementary Methods).

### Relative age estimation

To introduce biological meaning into age estimates of rhesus macaques and humans that have very different lifespans, as well as to overcome the inevitable skewing due to unequal distribution of data points from rhesus macaques and humans across age range, relative age estimation was made using the formula: Relative age = Age/maxLifespan where the maximum lifespan for rhesus macaques and humans were set to 42 years and 122.5 years, respectively. The maximum lifespan for the two species was chosen from the updated version of the *anAge* data base [23].

### Epigenome-wide association studies (EWAS) of age

EWAS was performed in each tissue separately using the R function “standardScreeningNumericTrait” from the “WGCNA” R package [41]. Next, the results were combined across tissues using Stouffer’s meta-analysis method. Our epigenome-wide association test studies of chronological age reveal that aging effects in one tissue are sometimes poorly conserved in another tissue.

#### Transcription factor enrichment and chromatin states

In our enrichment tests, we used the appropriate background comprised of CpGs that are represented on the mammalian array and that map to rhesus. Furthermore, we used an enrichment analysis software (GREAT) that properly adjusts for these and other potential sources of bias [42]. We evaluated our bioinformatics pipeline by uploading random sets of CpGs that did not result in significant enrichments [38].

The FIMO (Find Individual Motif Occurrences) program scans a set of sequences for matches of known motifs, treating each motif independently [43]. We ran TF motif (FIMO) scans of all probes on the HorvathMammalMethyl40 chip using motif models from TRANSFAC, UniPROBE, Taipale, Taipaledimer, and JASPAR data bases. A FIMO scan *p*-value of 1E-4 was chosen as cutoff (lower FIMO *p*-values reflect a higher probability for the local DNA sequence matching a given TF motif model). This cutoff implies that we find almost all TF motif matches that could possibly be associated with each site, resulting in an abundance of TF motif matches. We caution the reader that our hypergeometric test enrichment analysis did not adjust for CG content.

## Supplementary Information

Below is the link to the electronic supplementary material.

Supplementary file1 (DOCX 874 KB)
Supplementary file2 (XLSX 279 KB)

## Data Availability

The data will be made publicly available as part of the data release from the Mammalian Methylation Consortium. Genome annotations of these CpGs can be found on Github https://github.com/shorvath/MammalianMethylationConsortium.
