# Supplementary material for: Epigenetic clock and methylation studies in the rhesus macaque
Source: GeroScience. 2021 Sep 6;43(5):2441–53. doi: 10.1007/s11357-021-00429-8 (PMC8599607; doi:10.1007/s11357-021-00429-8)
Supplement: Supplementary file 1 — Supplementary file1 (DOCX 874 KB) Supplementary file2 (XLSX 279 KB) [file 11357_2021_429_MOESM1_ESM.docx]

**Supplementary Figures**

**Epigenetic clock and methylation studies in the rhesus macaque**

Steve Horvath^1,2#^, Joseph A. Zoller ^2#^, Amin Haghani^1^, Anna J. Jasinska ^3^, Ken Raj^4^, Charles E. Breeze^5^, Jason Ernst^6^, Kelli L. Vaughan^7^, Julie A. Mattison^7^

Correspondence:

1. Steve Horvath; E-mail: [shorvath@mednet.ucla.edu](mailto:shorvath@mednet.ucla.edu); Address: Gonda Building, 695 Charles Young Drive South, Los Angeles, CA 90095
2. Julie A. Mattison; Email: [Julie.mattison@nih.gov](mailto:Julie.mattison@nih.gov); Translational Gerontology Branch, National Institute on Aging, NIH.

**Supplementary Figures**

**Supplementary Figure 1. Unsupervised hierarchical clustering of tissue samples.**

Average linkage hierarchical clustering based on the interarray correlation coefficient (Pearson correlation). A height cut-off of 0.05 led to branch colors that largely correspond to Tissue type (second panel).

The second color band encodes tissue type: green=liver, red=lung, blue=blood, adipose=turquoise, brown=cerebral cortex, yellow=kidney, black=muscle, pink=skin. A handful of arrays are severe outliers (left- and right most clusters) as indicated by the third color band (turquoise samples are severe outliers). These technical outliers probably result from insufficient amounts of DNA. These outlying samples were removed from the analysis.

**
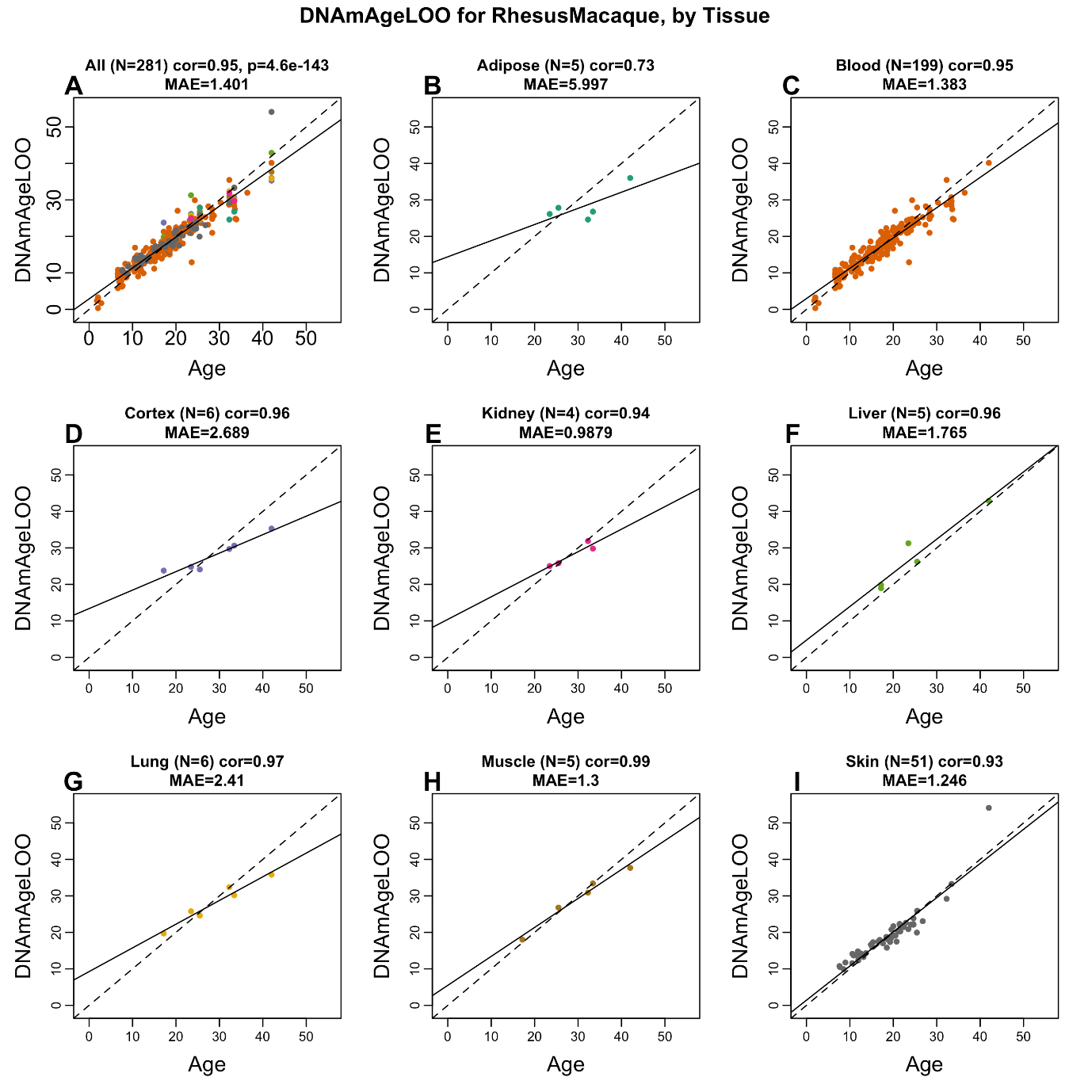
**

**Supplementary Figure 2: Pan-tissue clock for rhesus macaque applied to different tissues.** Each panel correlates the chronological age (x-axis) at the time of tissue collection with the leave-one-out (LOO) estimate of DNA methylation age. A) All tissues. Dots are colored by tissue type as indicated in the remaining panels. Results for B) adipose, C) blood, D) brain cortex, E) kidney, F) liver, G) lung, H) muscle, I) skin. Each panel reports the sample size (N), Pearson correlation, and the median absolute error, i.e., the median of the absolute difference between DNAmAgeLOO and chronological age. The dashed line is the diagonal y=x. The solid line corresponds to the least-squares regression line.

**
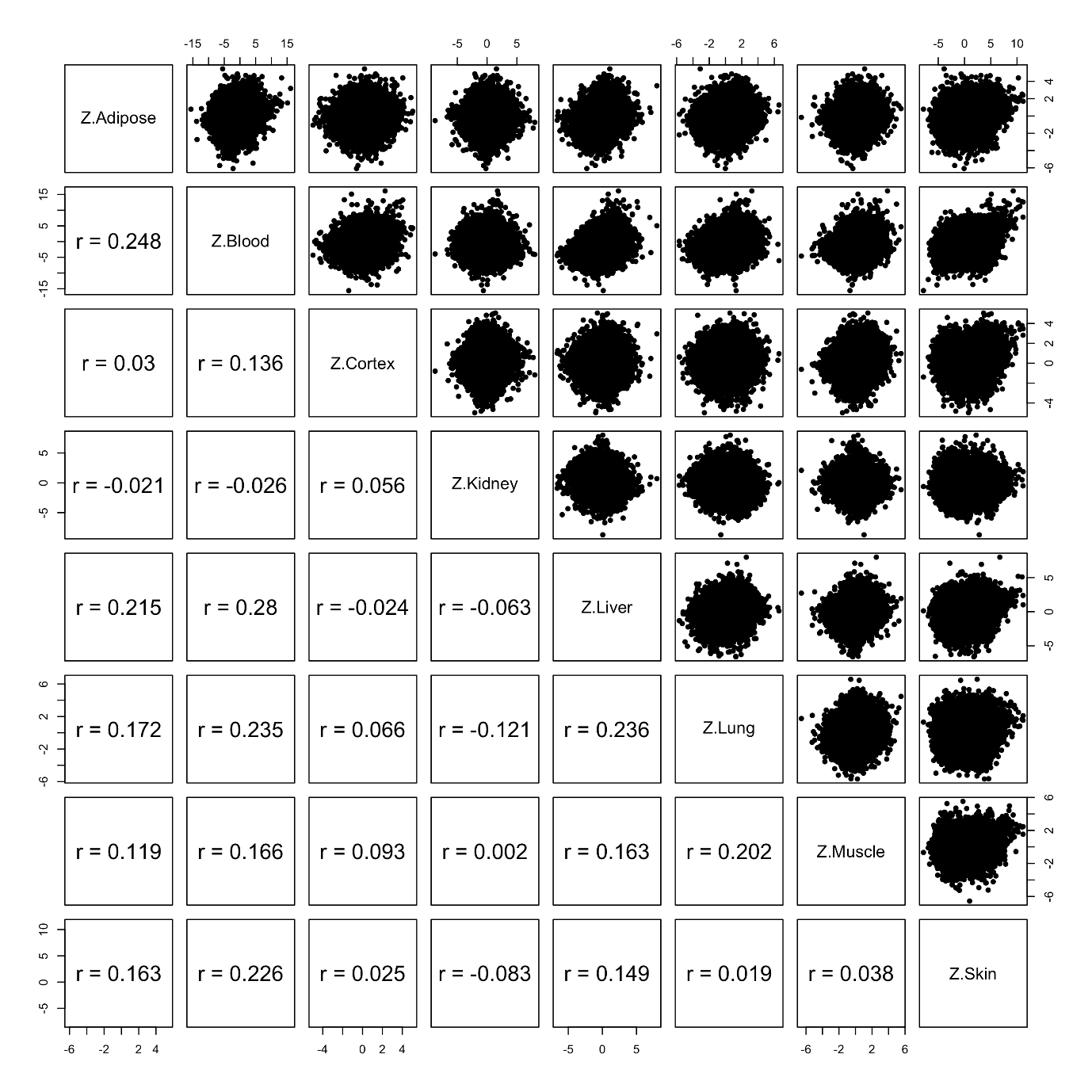
**

**Supplementary Figure 3**. EWAS was performed in each macaque tissue separately using the R function "standardScreeningNumericTrait" from the "WGCNA" R package. Z statistics from correlation tests in different tissues. Upper panels report scatter plots for Z statistics in different tissues. Lower panels: corresponding Pearson correlation coefficients.
